# Shortening antibiotic duration in the treatment of acute cholangitis: rationale and study protocol for an open-label randomized controlled trial

**DOI:** 10.1186/s13063-020-4046-4

**Published:** 2020-01-17

**Authors:** Kentaro Iwata, Asako Doi, Yuichiro Oba, Hiroo Matsuo, Kei Ebisawa, Manabu Nagata, Sho Nishimura, Kenichi Yoshimura, Atsuhiro Masuda, Hideyuki Shiomi, Yuzo Kodama

**Affiliations:** 1 Division of Infectious Diseases Therapeutics, Kobe University Graduate School of Medicine, Kusunokicho 7-5-2, Chuoku, Kobe, Hyogo 650-0017 Japan; 2 Department of Infectious Diseases, Kobe City Medical Center General Hospital, Minatojimaminamimachi, Chuoku, Kobe, Hyogo 650-0047 Japan; 3 Department of General Medicine, Osaka General Medical Center, Bandaihigashi 3-1-56, Sumiyoshi, Osaka, 558-8558 Japan; 4 Department of Infectious Diseases, Hyogo Prefectural Amagasaki General Medical Center, Higashinanbacho 2-17-77, Amagasaki, Hyogo 660-8550 Japan; 5 Innovative Clinical Research Center (iCREK), Kanazawa University Hospital, 13-1 Takara-machi, Kanazawa, Ishikawa 920-8641 Japan; 6 Department of Gastroenterology, Kobe University Graduate School of Medicine, Kusunokicho 7-5-2, Chuoku, Kobe, Hyogo 650-0017 Japan

**Keywords:** Acute cholangitis, Antimicrobial therapy, Short-course therapy, RCT

## Abstract

**Background:**

Antimicrobial therapy with appropriate biliary drainage is considered the standard of care for acute cholangitis, but the optimal duration of antimicrobial therapy remains unknown. Seven to 10 days of antimicrobial therapy are common for the treatment of acute cholangitis, but a recent retrospective cohort study suggested a shorter duration might be effective. A shorter duration of antimicrobial therapy can be beneficial in decreasing the length of hospital stay, improving patients’ quality of life, decreasing adverse effects, and even contributing to a decrease in the occurrence of antimicrobial resistance.

**Methods/design:**

We will conduct a multi-centre, open-label, randomized, non-inferiority trial to compare short-course therapy (SCT) with conventional long-course therapy (LCT) in treating patients with acute cholangitis. SCT consists of 5-day intravenous antimicrobial therapy if the patients had clinical improvement, while at least 7 days of intravenous antibiotics will be provided to the LCT group. The primary outcome is clinical cure at 30 days after onset. Patients will be randomly assigned in an open-label fashion. A total sample size of 150 was estimated to provide a power of 80% with a one-sided α level of 2.5% and a non-inferiority margin of 10%.

**Discussion:**

This trial is expected to reveal whether SCT is non-inferior to conventional LCT or not, and may provide evidence that one can shorten the treatment duration for acute cholangitis for the benefit of patients.

**Trial registration:**

University Hospital Medical Information Network, UMIN000028382. Registered on 30 August 2017.

## Background

Acute cholangitis is a common disorder which places a substantial burden on patients and the acute care system [1–4]. Antimicrobial therapy with appropriate biliary drainage is considered the standard of care [4, 5], but the optimal duration of antimicrobial therapy remains unknown. The commonly used treatment for acute cholangitis is 7–10 days of antimicrobial therapy [5], but a recent retrospective cohort study suggested a shorter duration might be equally effective [6]. A shorter duration of antimicrobial therapy can be beneficial in decreasing the length of hospital stay, improving patients’ quality of life, decreasing the adverse effects of antibiotics such as *Clostridioides difficile* infection, and even contributing to a decrease in the occurrence of antimicrobial resistance [7–10].

In this trial, we will compare antimicrobial therapy of a shorter duration with a conventional, longer duration, to investigate whether the short-course therapy (SCT) is non-inferior to conventional long-course therapy (LCT) in terms of clinical cure and other clinically important outcomes.

## Methods/design

### Design

**Fig. 1:**
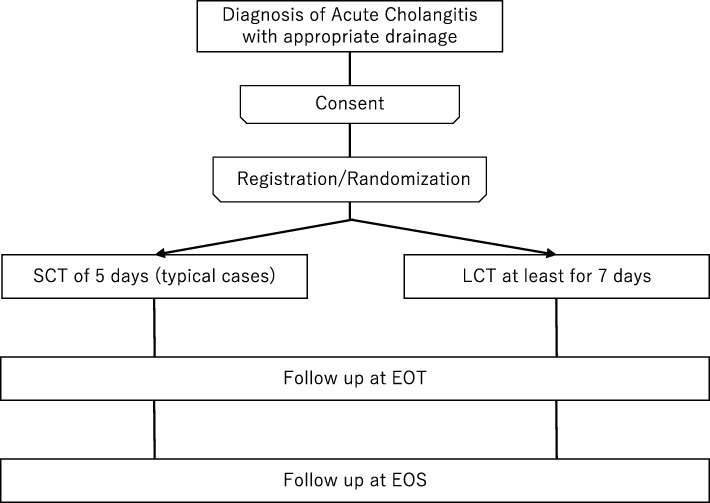
Study design. *SCT* short-course therapy, *LCT* long-course therapy, *EOT* end of treatment, *EOS* end of study

We will conduct a multi-centre, open-label, randomized, non-inferiority trial to compare SCT with conventional LCT in treating patients with acute cholangitis. The final trial report will follow the Consolidated Standards of Reporting Trials (CONSORT) statement and its extension to non-inferiority trials [11]. This study was registered at the University Hospital Medical Information Network under registry number UMIN000028382. The study protocol was written in accordance with the Standard Protocol Items: Recommendations for Interventional Trials (SPIRIT) guidelines (Additional file 1 shows the SPIRIT checklist). The flowchart of the study design is shown in Fig. 1.

### Setting

The study will be conducted at four tertiary referral hospitals in Japan (see Table 1).

**Table 1:** Participating institutions and investigators

Institution	Investigators
Kobe University Hospital	Kentaro Iwata, MD, PhD
Kobe City Medical Center General Hospital	Asako Doi, MD
Osaka General Medical Center	Yuichiro Oba, MD
Hyogo Prefectural Amagasaki General Medical Center	Hiroo Matsuo, MD

### Patients

Patients with acute cholangitis diagnosed by gastroenterologists based on findings such as fever, abdominal pain, liver function test abnormality, or imaging studies will be eligible for trial entry. If the infectious disease doctors are allowed to participate in the care of the patients, and if the treating physicians and the patients have agreed to participate in the trial, the patients are registered as potential study participants. The study participants are enrolled in this trial if they meet all of the inclusion criteria and none of the exclusion criteria.

### Inclusion criteria

The inclusion criteria are as follows:

1. Patients are 20 years or older.
2. They are diagnosed as having acute cholangitis by treating gastroenterologists.
3. Biliary duct obstruction was removed via procedures such as endoscopic retrograde cholangiopancreatography (ERCP), or there is no evidence of biliary duct obstruction by tests such as imaging studies to begin with.

### Exclusion criteria

The exclusion criteria are that:

1. The patient did not provide written informed consent.
2. Any biliary duct obstruction was not removed.
3. Treating physicians or the investigators judged that inclusion in the study was inappropriate.

The presence of bacteraemia is not an exclusion criterion.

### Ethics and informed consent

The clinical trial will be carried out according to the principles of the Declaration of Helsinki and Ethical Guidelines for Medical and Health Involving Human Subjects published by the Ministry of Health, Labour and Welfare of Japan and the Japanese Ministry of Education, Culture, Sports, Science and Technology. The study protocol was approved by the ethics committees of the participating hospitals. Written informed consent will be obtained from all patients or their representatives.

### Randomization and allocation concealment

Patients are randomly allocated to each treatment arm at a 1:1 ratio before or within 24 h after initiating antimicrobial therapy. Randomization will be performed using a stochastic minimization procedure centrally at the study centre (Division of Infectious Diseases Therapeutics, Kobe University Graduate School of Medicine). We will use an electronic data capture system to conduct randomization and data collection.

### Trial interventions

The antibiotics given to the study participants should be commercially available and approved for use in Japan. They will be used at the marketed accepted dosage as indicated in each package insert. The initial antibiotics will be selected at the discretion of either the treating physician or the consultant infectious disease doctor. Selected antibiotics can be changed to other antibiotics during the treatment based on culture/susceptibility tests results or potential adverse reactions that are suspected or occurred in the patient. Dose adjustments based on patients’ renal function or other criteria are performed as judged appropriate and necessary by the consultant infectious disease doctors.

**Fig. 2:**
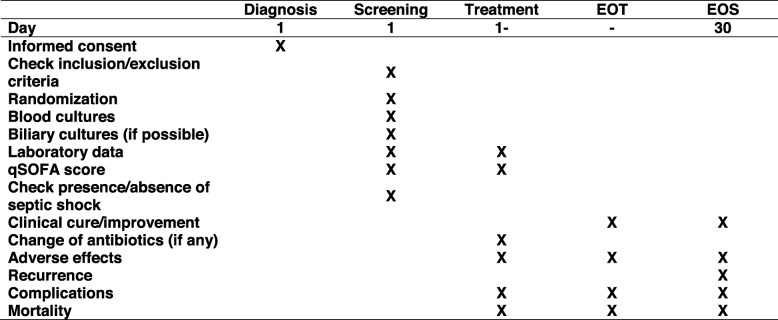
Schedule of assessments. *EOT* end of treatment, *EOS* end of study, *qSOFA* quick Sequential Organ Failure Assessment

In the SCT group, intravenous antibiotics will be continued at least for 5 days, and can be discontinued if all of the following criteria are fulfilled: (1) maintenance of body temperature under 37.8 °C for more than 48 h, (2) systolic blood pressure above 90 mmHg, (3) heart rate below 100 beats/min, (4) respiratory rate below 24 breaths/min, and oxygen saturation at room air above 90%. In the LCT group, intravenous antibiotics will be given for the duration of usual care, at least for 7 days, at the discretion of both the treating gastroenterologists and the consultant infectious disease doctors, provided that there are no biliary duct obstructions remaining, as stated in the inclusion/exclusion criteria. In the SCT group, positive blood culture results will not alter the duration of the treatment unless other complications which necessitate prolongation of the treatment, such as abscess or infective endocarditis, occur and either the treating physicians or the consultant infectious disease doctors remove the patient from the intervention. These patients will still be included in the analysis on an intention-to-treat (ITT) basis but will be excluded from the per-protocol analysis. The schedule of assessments is shown in Fig. 2.

### Assessment and follow-up

Clinical assessment is performed at baseline and daily throughout the study treatment, at the end of therapy (EOT), and at discharge from the hospital or 30 days after onset (end of study, EOS).

### Outcome measures

The primary outcome is clinical cure at 30 days after onset (EOS). Clinical cure is defined as disappearance of all clinical symptoms which were present upon diagnosis.

The secondary outcomes are clinical improvement after 30 days, mortality at day 30 after the diagnosis, or in-hospital mortality, occurrence of adverse effects, and recurrence or complications of acute cholangitis. Clinical improvement is defined as decrease but not disappearance of clinical symptoms which were present upon diagnosis.

### Sample size

The primary efficacy analysis will assess the non-inferiority of the clinical cure rate of SCT compared with LCT. The margin of non-inferiority is set at 10% on the statistically acceptable tolerance and clinically acceptable margin. This margin has been used as accepted in the field of infectious diseases [12, 13]. Because non-inferiority clinical trials in infectious diseases usually cannot rely on previous placebo controlled trials to calculate and estimate inferiority margins, we decided to use the rather arbitrary number of 10% as is conventionally employed. Regarding clinical cure, we judged the margin to likely be acceptable to most patients. The non-inferiority of SCT is concluded if the upper limit of the one-sided 97.5% confidence interval (CI) for the difference in clinical response (standard SCT) is less than 10%. To achieve a power of 80% with an α level of 2.5%, assuming as stated in the previous retrospective study a clinical cure rate of 95% with standard therapy with the same cure rate in SCT [6], with a non-inferiority margin of − 10%, 75 patients are required in each group.

### Statistical analysis

We will analyse data using both ITT and per-protocol analyses. The per-protocol analysis population will consist of all randomized patients who are not lost to follow-up and have no major protocol deviations. We will attest the non-inferiority of the primary outcome on the basis of the normal theory test for binomial proportions. We will conduct the primary analysis without adjustment of potential confounders.

Secondary outcomes will also be analysed under a non-inferiority assumption, as appropriate. Pre-defined subgroup analyses for the primary and secondary outcomes include (1) presence or absence of septic shock at diagnosis; (2) presence or absence of bacteraemia; (3) initial antibiotics covering or not covering causative organisms; (4) Gram-positive organisms causing cholangitis; and (5) quick Sequential Organ Failure Assessment (qSOFA) score.

Clinical outcomes are confirmed by the investigators either by calling the patients by telephone or seeing them at an outpatient clinic visit, or by checking the clinical chart if the patient is still hospitalized.

All *P* values are one-sided, and *P* < 0.025 is considered statistically significant. All statistical analyses will be performed using STATA version 15.0 (StataCorp, College Station, TX, USA), and R version 3.5.1 (the R Foundation for Statistical Computing, Vienna, Austria).

### Trial oversight

The trial will be managed by the Division of Infectious Diseases, Kobe University Hospital, Kobe, Japan. The data centre is at the same location, and the data managers will centrally monitor the data during the study period. A Steering Committee was involved in protocol development and will oversee study progress (Table 2). We will not have a specific data and safety monitoring board, but the Steering Committee will perform an interim analysis to ensure the safety and efficacy of the trial therapy and will monitor the integrity and validity of the data collected and the conduct of the clinical trial. The data management team will report to the Steering Committee monthly with the numbers of patients registered. The data management team will also report mortality and occurrence of serious adverse events immediately to the committee.

**Table 2:** Study oversight

Role in study	Name	Institution
Principal investigator	Kentaro Iwata	Division of Infectious Diseases, Kobe University Hospital
Steering Committee	Asako Doi	Department of Infectious Diseases, Kobe City Medical Center General Hospital
Steering Committee	Yuichiro Oba	Department of General Medicine, Osaka General Medical Center
Steering Committee	Hiroo Matsuo	Department of Infectious Diseases, Hyogo Prefectural Amagasaki General Medical Center
Data management	Kei Ebisawa	Division of Infectious Diseases, Kobe University Hospital
Data management	Sho Nishimura	Division of Infectious Diseases, Kobe University Hospital
Data management	Manabu Nagata	Division of Infectious Diseases, Kobe University Hospital
Event Adjudication Committee	Atsuhiro Masuda	Department of Gastroenterology, Kobe University Graduate School of Medicine
Event Adjudication Committee	Hideyuki Shiomi	Department of Gastroenterology, Kobe University Graduate School of Medicine
Event Adjudication Committee	Yuzo Kodama	Department of Gastroenterology, Kobe University Graduate School of Medicine
Study statistician	Kenichi Yoshimura	Innovative Clinical Research Center (iCREK), Kanazawa University Hospital
Study secretariat	–	Division of Infectious Diseases, Kobe University Hospital
Project management	–	Division of Infectious Diseases, Kobe University Hospital

## Discussion

The current trial will examine whether SCT for acute cholangitis with appropriate biliary duct drainage is not inferior to conventional LCT. A retrospective cohort study with propensity score analysis suggested that the efficacy of SCT as well as occurrence of complications are similar to those of LCT [6]. If non-inferiority was achieved, SCT has several advantages over LCT in terms of length of hospital stay, potential adverse effects from antimicrobial therapy, cost, and emergence of antimicrobial resistance [7–10].

The current proposed trial has some inherent limitations. First, for practical reasons, we were not able to design the study to be double-blinded. Both patients and investigators will know to which group the patients belong. However, we consider that the outcomes we set are obvious and would not be likely to be impaired significantly by the trial being an open-label design, as demonstrated in a previous similar study [14]. Second, since clinical outcomes will be judged by our investigators, there is an inherent issue of potential subjectivity on outcome judgement. On the other hand, based on the previous study with a very high cure rate, we believe this is a relatively small problem unlike treatments of diseases with a lower success rate. In addition, it is not practical to employ independent investigators in multi-centre settings. Third, because this study is conducted solely in Japan, although it is a multi-centre trial, the results of the study might not be applicable in different settings outside the country. The results of the proposed study are not applicable to those patients with acute cholangitis when bile duct obstruction remains.

To the best of our knowledge, this will be the first trial to answer the clinical question of whether shortening of antimicrobial therapy for the treatment of acute cholangitis is possible without impairing treatment safety and efficacy. We hope that the trial aids in establishing a novel therapy to optimize the duration of antibiotic therapy for this rather common disease.

### Trial status

The protocol number is version 1.3 dated September 21, 2018. Patient recruitment began on December 19, 2018. We recruited the first patient on June 27, 2019. The trial is scheduled to end on December 31, 2021.

## Supplementary information

**Additional file 1.** SPIRIT checklist.

## Data Availability

The datasets used and/or analysed during the current study are available from the corresponding author upon reasonable request.

## Abbreviations

EOS: End of study
EOT: End of treatment
ERCP: Endoscopic retrograde cholangiopancreatography
LCT: Long-course therapy
qSOFA: Quick Sequential Organ Failure Assessment
RCT: Randomized controlled trial
SCT: Short-course therapy
